# Output speed control for hydro-mechanical continuously variable transmission of tractor

**DOI:** 10.1371/journal.pone.0308493

**Published:** 2024-09-24

**Authors:** Zhiqiang Xi, Ziying Luo, Fuyi Cao, Lianbo Niu, Liyou Xu

**Affiliations:** 1 College of Vehicle and Traffic Engineering, Henan University of Science and Technology, Luoyang, China; 2 State Key Laboratory of Intelligent Agricultural Power Equipment, Luoyang, China; 3 School of Mechanical and Electronically Engineering, Xinxiang University, Xinxiang, China; HNBGU: Hemvati Nandan Bahuguna Garhwal University, INDIA

## Abstract

In order to improve the speed stability of tractors equipped with hydro-mechanical continuously variable transmission (HMCVT) during field operations, the speed regulation characteristics, torque characteristics, and power diversion characteristics of HMCVT are analyzed. And a comprehensive control strategy for HMCVT output speed is proposed by analyzing the factors affecting the fluctuation of output speed, which combines engine speed control and displacement ratio control of hydraulic speed control system. This strategy adopts fuzzy control to regulate the engine speed to maintain the stability of engine operation. Moreover, adopting feed forward compensation control for speed compensation enhances the anti-interference ability of the hydraulic speed control system. And combined with model predictive control, adjust the swash plate swing angle of the variable pump to reduce the fluctuation of HMCVT output speed. By establishing joint simulation model, the effectiveness of the control strategy was verified using tractor acceleration mode and step load disturbance mode. The results show that the strategy can reduce the fluctuation rate of engine speed and impact degree, shorten the adjustment time, and improve the stability of tractor operating speed.

## Introduction

The main function of the tractor is to be used in conjunction with various traction and driving machines to complete agricultural field operations [1]. The transmission performance of the gearbox will have an important impact on the tractor and its transmission system.

Hydro-mechanical continuously variable transmission (HMCVT) is a type of dual power flow continuously variable transmission that combines hydraulic power flow and mechanical power flow to transmit power [2, 3]. Compared with the single transmission path of the traditional CVT, the dual transmission path of the HMCVT has more advantages. HMCVT not only retains the characteristics of stepless speed regulation, but also has much higher efficiency than standard CVT. Hence, the HMCVT has been become widely used in agricultural tractors [4–6].

In recent years, many literature have conducted on research of HMCVT. Regarding mechanical characteristic analysis for HMCVT, Xia et al. [7] proposed an optimized design method for the selection of the structural parameters of the power-cycle HMCVT to ensure competent overall performance. Li et al. [8] analyzed the working principle of HMCVT and successfully developed a hydraulic mechanical stepless gearbox suitable for highpower tractors for both water and drought. Wu et al. [9] investigated a HMCVT for the all-terrain vehicle with single planetary and input differential. Wan et al. [10] establishes the general characteristic equations for the input coupling type of HMCVT based on the wheel loader and determines reasonable structural forms for connection of working conditions. He et al. [11] studied power distribution in hydraulic and mechanical transmission lines of the virtual prototype of the HMCVT. Some other literature focused on the shift strategy of HMCVT. Zhu et al. [12, 13] obtained the design parameter of HMCVT through the analysis of kinematics and dynamics for the big horsepower tractor and studied the shift strategy of HMCVT based on the physical parameters and shift time. Li et al. [14] investigated a comprehensive shifting control strategy of a HMCVT in a high horsepower tractor for enhancing shift quality. To reduce impact during the shift and improve engagement quality, Lu et al. [15] analyzed the influence of system oil pressure and the clutch’s working flow on the engagement characteristics of the wet clutch in terms of shift quality. Zhao et al. [16] established the dynamic model of HMCVT based on Simulation X, introduced the friction coefficient into the wet clutch model, and analyzed the influence of control oil pressure on the sliding friction power and peak torque of wet clutches during HMCVT shifting. To decrease shift impact on agricultural tractors with HMCVT and find the best working point, Qian et al. [17] conduct all-factor method and response surface method to design and carry out bench test of shift impact. In order to optimize speed ratio follow-up control effects of HMCVT, Yu et al. [18] designed a variable universe fuzzy PID controller based on the expansions of quantizing factors and decision-making factors, which presents better control effectiveness and dynamic quality.

In addition, Wang et al. [19] established an HMCVT model based on Simulation X, calibrated the model according to experimental data, and then built the efficiency model. Cheng et al. [20] studied the efficiency model of the hybrid continuously variable transmission based on the improved simulated annealing algorithm. Zhang et al. [21] provides a method to analyze the overall efficiency of HMCVT. Aiming at the problems of accurate method, low accuracy, and high noise in the prediction of HMCVT transmission efficiency, Lu et al. [22] proposes a method based on variational mode decomposition, particle swarm optimization, and back propagation neural networks to improve the quality of transmission efficiency prediction.

From the analysis above, we can see that scholars’ research on HMCVT is focused on the structure, shifting strategy and efficiency characteristics, but there is little research on the output speed control. By controlling the output speed, HMCVT can quickly recover to a stable speed under input load changes or external disturbances. This not only improves the efficiency and accuracy of tractor operations, but also conforms to the development direction of global precision agriculture.

This article aims to enhance the stability of output speed, analyze the working principle and characteristics of HMCVT, and propose a comprehensive control strategy based on fuzzy control algorithm, feedforward compensation control, and model predictive control. This strategy can reduce the dynamic time delay of the hydraulic speed control system, improve its response speed, and mitigate the issue of transmission output speed fluctuations. Compared with PID control algorithm, this strategy exhibits better tracking performance, robustness, and anti-disturbance ability.

## Analysis of HMCVT characteristic

### Structure of HMCVT

**Fig 1:**
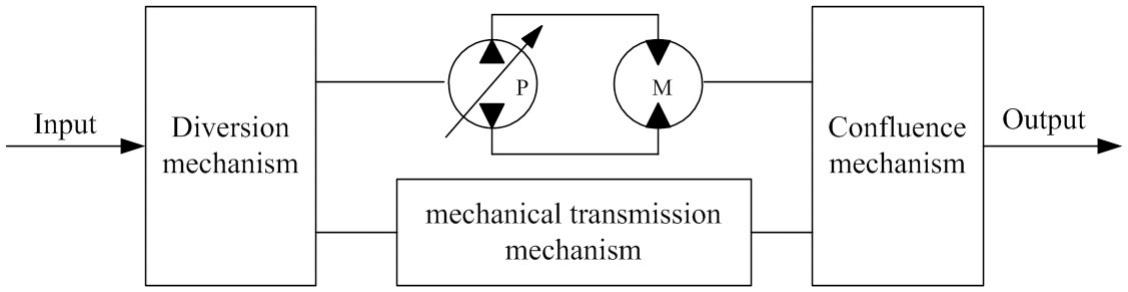
Structure principle of the HMCVT.

As shown in Fig 1, HMCVT consists of mechanical transmission mechanism, pump-motor hydraulic continuously variable transmission system, planetary gear mechanism for power diversion and convergence, and other parts. When the transmission ratio of the mechanical transmission mechanism is determined, adjusting the transmission ratio of the hydraulic continuously variable transmission device can cause the HMCVT transmission ratio to change continuously within a certain range, thereby enabling power to be output through diversion, speed change, and convergence, achieving high-power and efficient continuously variable transmission.

When a wide speed range is required, due to the limited transmission range of the hydraulic speed control device, it is often necessary for the mechanical transmission mechanism to shift gears or change the transmission structure through the engagement and separation of the clutch or brake, so as to expand the range of HMCVT transmission ratio and form a multi-stage HMCVT with continuous transmission ratio changes.

**Fig 2:**
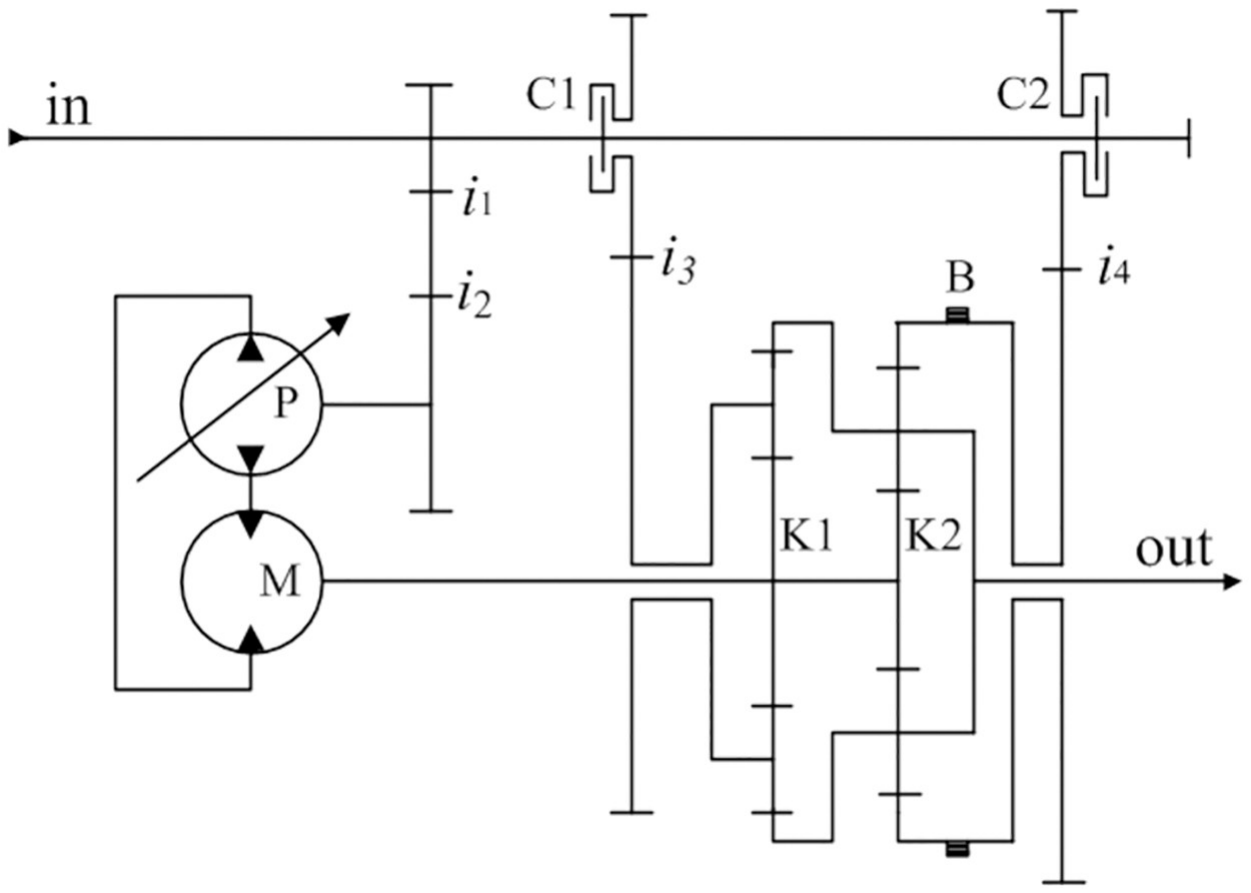
Three-stage HMCVT structure.

Taking into account the working conditions and transmission structure of tractor, the three-stage HMCVT structure studied in this paper is shown in Fig 2. The transmission mainly consists of a mechanical transmission system, hydraulic speed control system (consisting of a variable displacement pump P and a fixed displacement motor M), a dual planetary gear set (K1 and K2), and range-shift actuator composed of clutches (C1 and C2) and brake B. The transmission ratios of the various gear pairs are designated as *i*_1_, *i*_2_, *i*_3_, and *i*_4_.

The working mode of the HMCVT can be changed by controlling the engagement and separation status of the range-shift actuator. It has three working modes: pure hydraulic transmission mode (H), hydraulic mechanical mode 1 and mode 2 (HM1 and HM2). Table 1 shows the range-shift actuator status of the HMCVT under different working modes.

**Table 1:** Status of range-shift actuator in different modes.

Range-shift actuator	H	HM1	HM2
C_1_		√	
C_2_			√
B	√		

**Fig 3:**
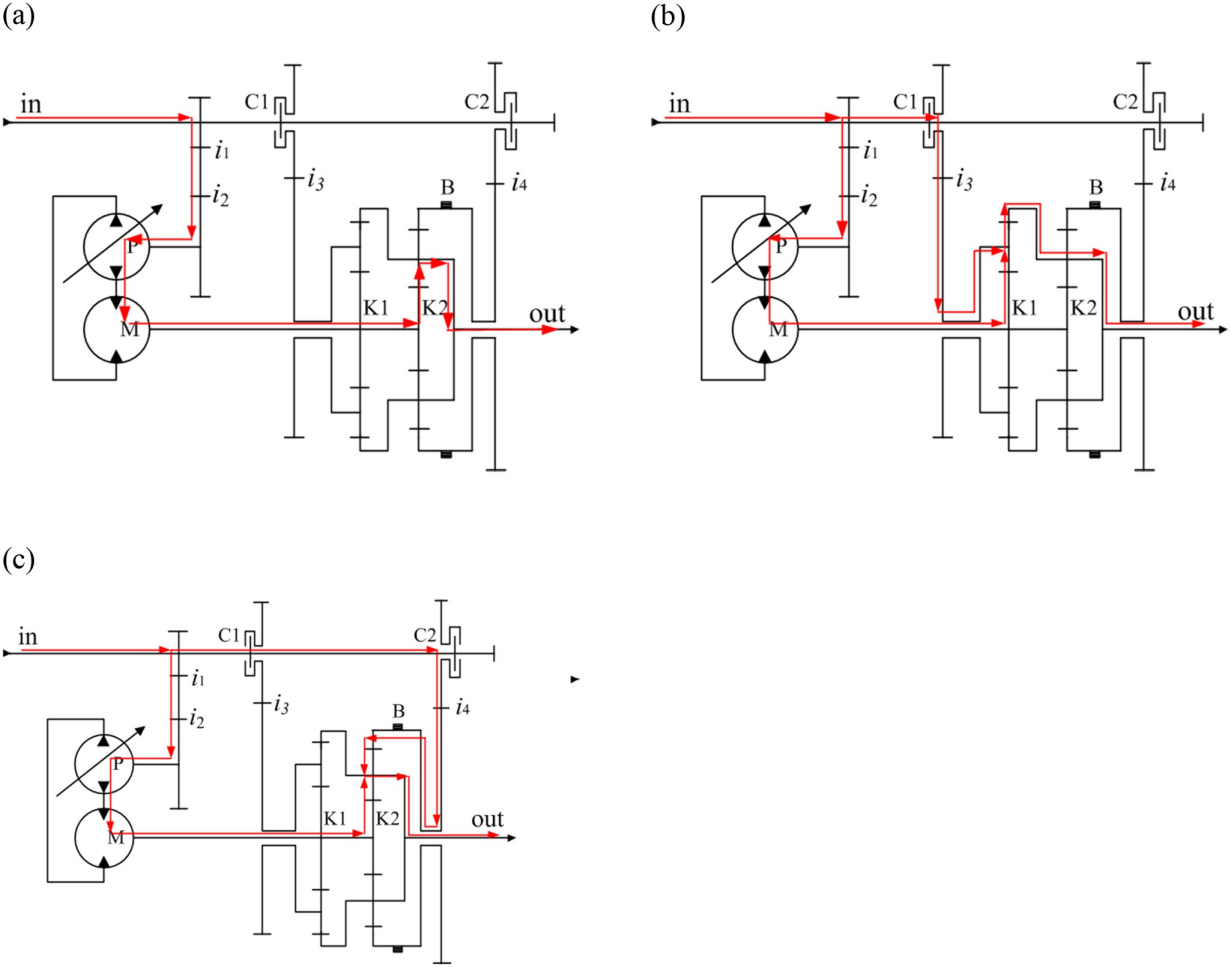
Power transmission route of different mode. (a) H-mode; (b) HM1 mode; (c) HM2 mode.

Fig 3 illustrate the power transmission routes of the HMCVT when it is operating in the H, HM1, and HM2 modes, respectively.

### HMCVT characteristics

#### 1. Speed regulation characteristics

By adjusting the displacement ratio of the hydraulic speed control system, the HMCVT can achieve stepless speed control function within a specific range. The speed regulation characteristics of it are reflected in the relationship between its speed ratio *i* and the displacement ratio *e* of the hydraulic speed control system.

$$i=\frac{n_{\text{out}}}{n_{\text{in}}}$$ (1)

$$e=\frac{D_{\text{p}}}{D_{\text{pmax}}}$$ (2)

Where *n*_out_ and *n*_in_ are the speed of output shaft and input shaft of HMCVT; *D*_*p*_ and *D*_*pmax*_ are the actual displacement and the maximum displacement of the variable displacement pump.

Without considering the transmission efficiency of the hydraulic speed control system, there is a specific relationship between the speed and displacement of the variable displacement pump and the fixed-displacement motor:

$$D_{\text{p}}n_{\text{p}}=D_{\text{m}}n_{\text{m}}$$ (3)

Where *D*_m_ is the actual displacement of the fixed-displacement motor, *n*_p_ is the rotational speed at the input end of the variable displacement pump, *n*_m_ is the rotational speed at the output end of the motor.

*(1) Hydraulic mode (H)*. In the pure hydraulic mode, both clutches C1 and C2 are in the separated state and do not participate in power transmission, while the brake B is in the operating state.

The input rotational speed of the variable displacement pump is:

$$n_{\text{p}}=\frac{n_{\text{in}}}{i_{1}i_{2}}$$ (4)

The output rotational speed of the fixed-displacement motor is:

$$n_{\text{m}}=en_{\text{p}}$$ (5)

The speed relationship among the three components of planetary gear set K2 is as follows:

$$n_{\text{s2}}+k_{\text{2}}n_{\text{r2}}-(1+k_{2})n_{\text{c2}}=0$$ (6)

Where *n*_s2_, *n*_*c*2_ and *n*_*r*2_ correspond to the rotational speeds of the sun gear, planet carrier, and ring gear of planetary gear set K2, respectively; *k*_2_ represents the characteristic parameter of planetary gear set K2.

The output shaft speed of the fixed-displacement motor is connected to the sun gear of the planetary gear set K2, therefore:

$$n_{s2}=n_{\text{m}}$$ (7)

The planet carrier of planetary gear set K2 is connected to the output shaft:

$$n_{\text{o-H}}=n_{\text{c2}}$$ (8)

Where *n*_o-H_ represents the speed of the output shaft of the HMCVT in H mode.

When the HMCVT operates in H mode, the ring gear of planetary gear set K2 is completely locked by brake B, thus:

$$n_{\text{r2}}=0$$ (9)

By combining Eqs 4–9, the speed ratio of the HMCVT operating in H mode can be derived as follows:

$$i_{\text{H}}=\frac{e}{(1+k_{2})i_{1}i_{2}}$$ (10)

Where *i*_H_ represents the speed ratio of the HMCVT operating in H mode.

*(2) Hydraulic mechanical mode 1 (HM1)*. When HMCVT operates in hydraulic mechanical mode 1, clutch C1 is engaged, and clutch C2 and brake B are not working. At this point, the input power is divided into hydraulic branch and mechanical branch. The hydraulic branch power reaches the sun gear of planetary gear set K1, while the mechanical branch power reaches the planet carrier of planetary gear set K1. These two branches of power couple at planetary gear set K1, and the combined power is then transmitted to the output shaft of the HMCVT by the gear ring.

Based on the characteristics of planetary gear sets, the speed relationship among the three components of planetary gear set K1 can be derived as follows:

$$n_{\text{s1}}+k_{1}n_{\text{r1}}-(1+k_{1})n_{\text{c1}}=0$$ (11)

Where *n*_s1_, *n*_r1_ and *n*_c1_ correspond to the rotational speeds of the sun gear, ring gear, and planet carrier of planetary gear set K1, respectively. *k*_1_ represents the characteristic parameter of planetary gear set K1.

In HM1 mode, when clutch C1 is engaged and transmitting power, the rotational speed of the planet carrier of planetary gear set K1 can be derived as follows:

$$n_{\text{c1}}=\frac{n_{\text{in}}}{i_{3}}$$ (12)

Since the fixed-displacement motor is connected to the sun gear of planetary gear set K1, thus:

$$n_{\text{m}}=n_{\text{s1}}$$ (13)

Due to the connection between the output shaft of HMCVT, the planet carrier of planetary gear set K2, and the ring gear of planetary gear set K1:

$$n_{\text{o-HM1}}=n_{\text{r1}}$$ (14)

Where *n*_o-HM1_ represents the speed of the output shaft of the HMCVT in HM1 mode.

By combining Eqs 4, 5 and 11–14, the speed ratio of the HMCVT operating in HM1 mode can be derived as follows:

$$i_{\text{HM1}}=\frac{\text{1}}{k_{1}}[\frac{1+k_{1}}{i_{3}}-\frac{e}{i_{1}i_{2}}]$$ (15)

Where *i*_HM1_ represents the speed ratio of the HMCVT in HM1 mode.

*(3) Hydraulic mechanical mode 2 (HM2)*. When HMCVT operates in hydraulic mechanical mode 2, both clutch C1 and brake B are disengaged, while clutch C2 is engaged. The input power is divided into hydraulic branch and mechanical branch. The hydraulic branch power reaches the sun gear of planetary gear set K2, while the mechanical branch power reaches the ring gear of planetary gear set K2. These two branches of power couple at planetary gear set K1, and the combined power is then transmitted to the output shaft of the HMCVT through the planet carrier.

In HM2 mode, the rotational speed of the ring gear of planetary gear set K2 can be derived as follows:

$$n_{\text{r1}}=\frac{n_{\text{in}}}{i_{4}}$$ (16)

By combining Eqs 4–8 and 15, it can be concluded that the speed ratio of HMCVT operating at HM2 mode is:

$$i_{\text{HM2}}=\frac{1}{1+k_{2}}\left[\frac{e}{i_{1}i_{2}}+\frac{k_{2}}{i_{4}}\right]$$ (17)

Where *i*_HM2_ represents the speed ratio of the HMCVT in HM2 mode.

**Fig 4:**
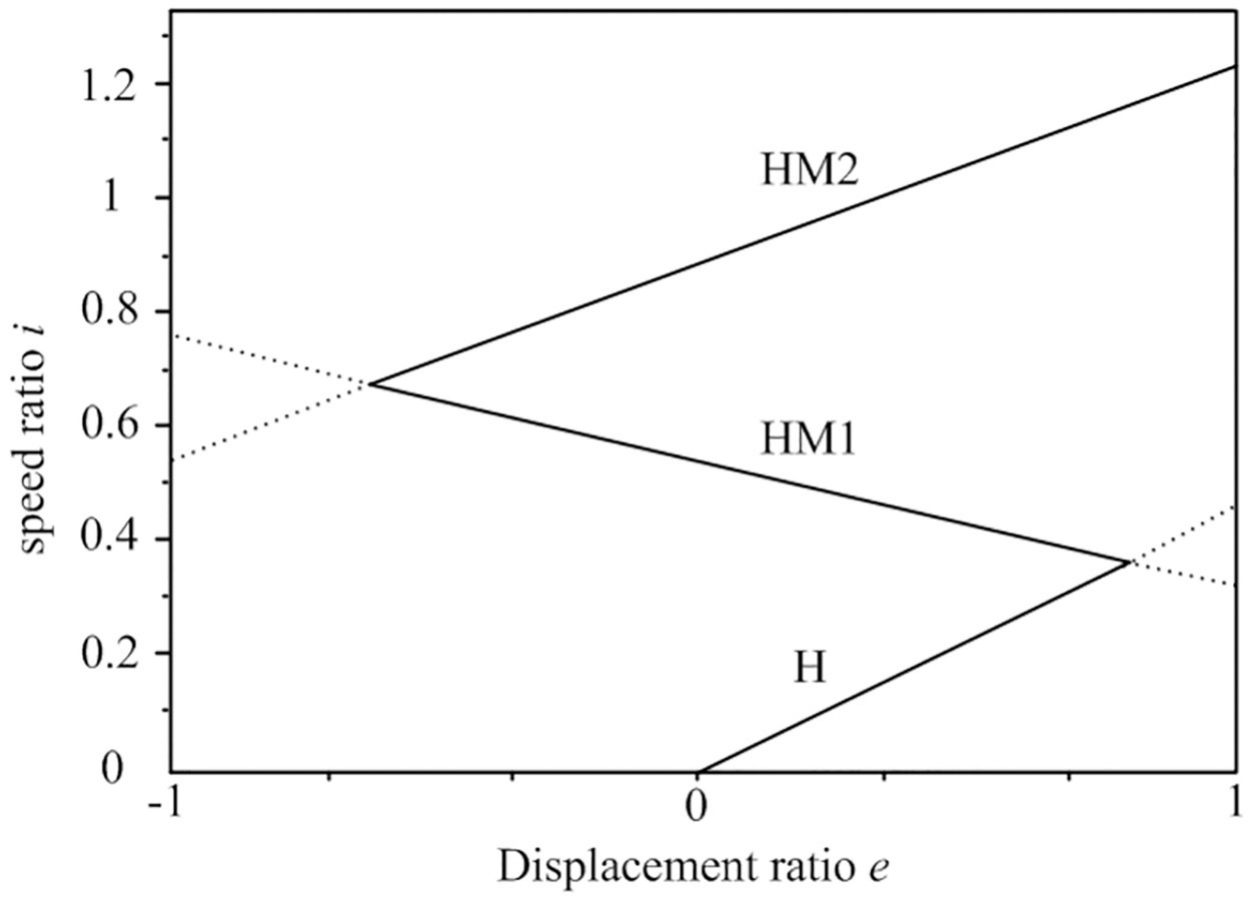
Characteristic curve of HMCVT speed regulation.

Based on Eqs 10, 15 and 17, the speed regulation characteristic curves of the HMCVT under different working modes, as shown in Fig 4, can be obtained. As the displacement ratio of the hydraulic speed control system changes, the speed ratio of HMCVT continuously increases from zero. This characteristic indicates that the tractor equipped with this HMCVT possesses continuous speed control ability starting from zero, enabling stepless speed control.

#### 2. Torque characteristics

The torque characteristic mainly describes the ratio between the HMCVT output shaft torque *T*_*o*_ and the motor output end torque *T*_*m*_. During the transmission process, the relationship between the torques of three components in the planetary gear set is as follows:

$$T_{si}:T_{ri}:T_{ci}=1:k_{i}:(1+k_{i})$$ (18)

Where *T*_*si*_, *T*_*ri*_ and *T*_*ci*_ correspond to the torque of the sun gear, ring gear, and planet carrier of planetary gear set Ki, respectively; *i* represents the planetary gear set number, *i* = 1 or 2;

*(1) H mode*. When the HMCVT operates in the pure hydraulic mode, the input power is transmitted to the planetary gear set K2 through the hydraulic speed control system. The output shaft of the HMCVT is connected to its planet carrier, thus:

$$T_{\text{o-H}}=T_{\text{c2}}$$ (19)

Where *T*_o-H_ represents the torque of the output shaft of the HMCVT in H mode.

The fixed-displacement motor is connected to the sun gear of the planetary gear set K2, therefore:

$$T_{\text{m}}=T_{\text{s2}}$$ (20)

Where *T*_m_ is the torque at the output end of the fixed-displacement motor.

Using Eqs 18–20, the torque ratio of the HMCVT in H mode can be derived as:

$$\left|\frac{T_{\text{o-H}}}{T_{\text{m}}}\right|=1+k_{2}$$ (21)

*(2) HM1 mode*. When the HMCVT operates in the hydraulic-mechanical mode 2, the ring gear of the planetary gear set K1 is connected to the planet carrier of the planetary gear set K2, and the output shaft of the HMCVT is connected to the planet carrier of the planetary gear set K2. Therefore, it can be derived that:

$$T_{\text{o-HM1}}=T_{\text{r1}}$$ (22)

Where *T*_o-HM1_ represents the torque of the output shaft of the HMCVT in HM1 mode.

The fixed-displacement motor is connected to the sun gear of the planetary gear set K1, which leads to:

$$T_{\text{m}}=T_{\text{s1}}$$ (23)

Using Eqs 18, 22 and 23, the torque ratio of the HMCVT in HM1 mode can be derived as:

$$\left|\frac{T_{\text{o-HM1}}}{T_{\text{m}}}\right|=k_{1}$$ (24)

*(3) HM2 mode*. When HMCVT operates in hydraulic mechanical mode 2, after the input power is split, it is coupled and output by planetary gear K2. At this time, the torque ratio of HMCVT is the same as that in H mode.

Based on the above analysis, it can be seen that the torque ratio of the HMCVT remains constant under different modes. The maximum output torque of HMCVT is limited by the output torque of fixed-displacement motor. By selecting an appropriate motor, it can be ensured that the torque characteristics of the HMCVT meet the requirements of the maximum power transmission of the engine.

#### 3. Power shunt characteristics

The power shunt ratio of the HMCVT refers to the ratio of the power transmitted through the hydraulic branch to the total power. Since the efficiency of hydraulic transmission is lower than mechanical transmission, the transmission efficiency of HMCVT will increase as the hydraulic power shunt ratio decreases. The power shunt ratios of the HMCVT under different modes are as follows:

*(1) H mode*. In the pure hydraulic mode, all the power is transmitted through the hydraulic branch. Therefore, the hydraulic power shunt ratio of the HMCVT in H mode is:

$$\rho_{\text{H}}=1$$ (25)

Where *ρ*_H_ represents the power shunt ratio of the HMCVT in the H mode.

*(2) HM1 mode*. In the HM1 mode, power is transmitted through the hydraulic branch and the mechanical branch. The hydraulic power shunt ratio under this mode is:

$$\rho_{\text{HM1}}=\left|\frac{P_{\text{H}}}{P_{\text{o-HM1}}}\right|=\left|\frac{T_{\text{m}}n_{\text{m}}}{T_{\text{o-HM1}}n_{\text{o-HM1}}}\right|=\left|\frac{T_{\text{s1}}n_{\text{s1}}}{T_{\text{r1}}n_{\text{r1}}}\right|$$ (26)

Where *ρ*_HM1_ represents the power shunt ratio of the HMCVT in HM1 mode; *P*_*H*_, *P*_*o-HM*1_ are the power transmitted through the hydraulic branch and the total transmission power of the HMCVT, respectively.

Using Eq 18, can derive:

$$\frac{T_{\text{s1}}}{T_{\text{r1}}}=\frac{1}{k_{1}}$$ (27)

Using Eqs 4, 5 and 13–15, can derive:

$$\frac{n_{\text{s1}}}{n_{\text{r1}}}=\frac{e}{i_{1}i_{2}i_{\text{HM1}}}$$ (28)

Combining the above equations, the power shunt ratio of the HMCVT in HM1 mode can be derived as:

$$\rho_{\text{HM1}}=\frac{ei_{3}}{(1+k_{1})i_{1}i_{2}-ei_{3}}$$ (29)

*(3) HM2 mode*. In the HM2 mode, both the hydraulic branch and the mechanical branch jointly undertake the task of transmitting power. The power shunt ratio under this mode is:

$$\rho_{\text{HM2}}=\left|\frac{P_{\text{H}}}{P_{\text{o-HM2}}}\right|=\left|\frac{T_{\text{m}}n_{\text{m}}}{T_{\text{o-HM2}}n_{\text{o-HM1}}}\right|=\left|\frac{T_{\text{s2}}n_{s1}}{T_{\text{c2}}n_{\text{r1}}}\right|$$ (30)

Where *ρ*_HM2_ represents the power shunt ratio of the HMCVT in HM2 mode, and *P*_o-HM2_ denotes the total transmission power in HM2 mode.

Using Eq 18, it can obtain:

$$\frac{T_{\text{s2}}}{T_{\text{c2}}}=\frac{1}{1\text{+}k_{\text{2}}}$$ (31)

By combining the Eqs 17 and 31, the power shunt ratio of the HMCVT in HM2 mode can be derived as follows:

$$\rho_{\text{HM2}}=\frac{ek_{1}i_{3}}{(1+k_{1})\left(1\text{+}k_{2}\right)i_{1}i_{2}-\left(\text{1+}k_{2}\right)ei_{3}}$$ (32)

**Fig 5:**
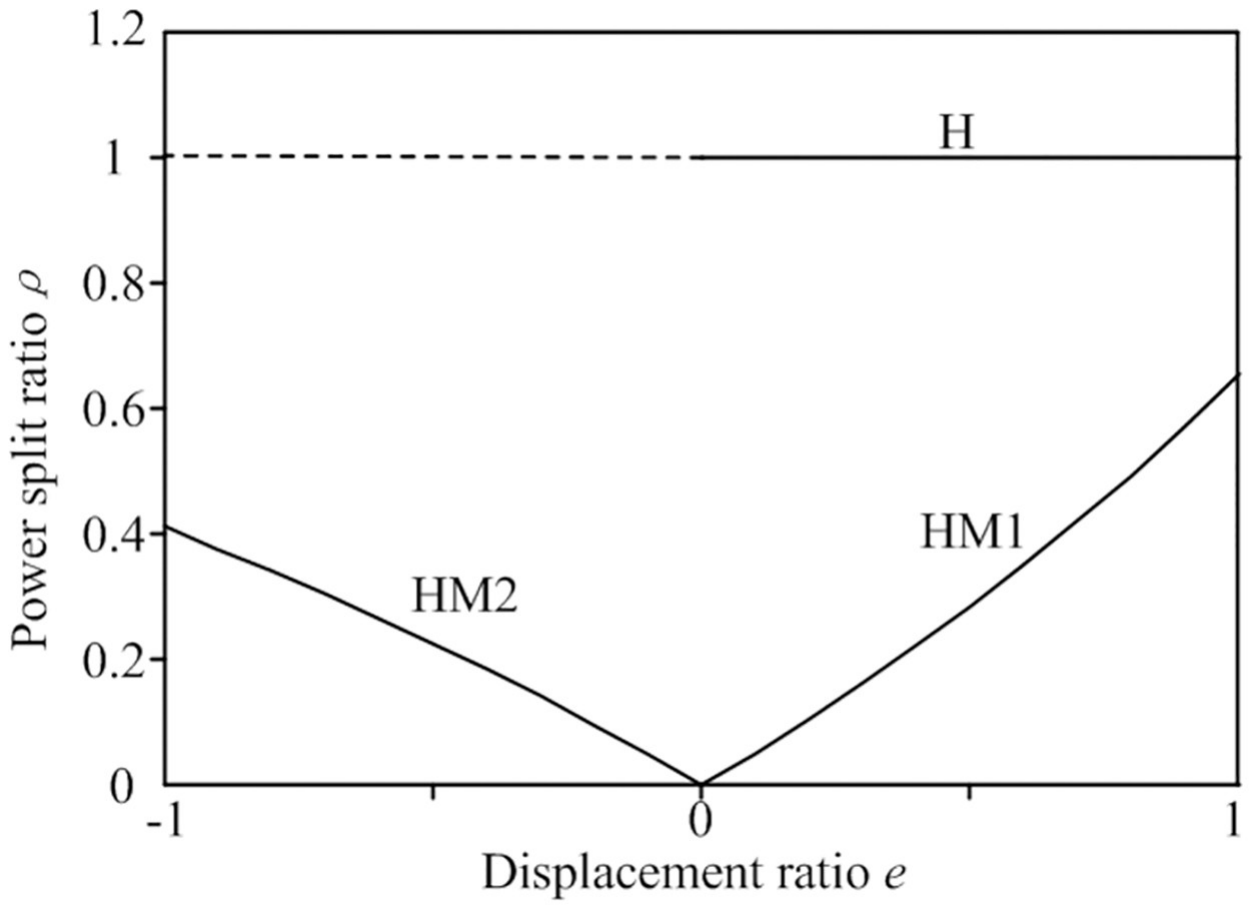
Characteristic curve of hydraulic power shunt ratio.

Based on Eqs 25, 29 and 32, the power shunt ratio characteristic curve can be plotted as shown in Fig 5. As observed from the figure, when the HMCVT operates in the hydraulic-mechanical modes HM1 and HM2, the hydraulic speed control system only transmits a portion of the power, ensuring the transmission efficiency of the tractor.

## Control strategy of output speed

By combining the model of the hydraulic speed-regulating system with the engine model, a comprehensive control strategy for the output speed of the tractor’s HMCVT is developed, focusing on controlling the displacement ratio of the hydraulic speed control system and the engine speed.

### Control of engine speed

#### 1. Control strategy for engine speed

**Fig 6:**
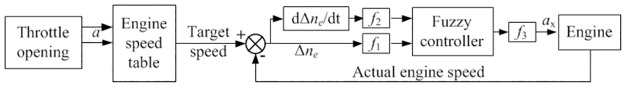
Engine speed control strategy.

As a high-order, nonlinear and large inertia complex system, the engine is often affected by various disturbances and uncertainties during its operation. Traditional control theories are difficult to meet its high-performance requirements. Therefore, this article chooses fuzzy control to control the engine speed during the HMCVT speed regulation process, and the control strategy is shown in Fig 6. The core of this strategy is to achieve flexible control between the accelerator pedal and throttle opening. The accelerator pedal is mainly used to reflect the driver’s driving intention, while the throttle opening is independently controlled by a fuzzy controller. To ensure that the tractor has sufficient power in various operating scenarios, the engine speed data at the maximum output torque of the engine at each throttle opening is stored in the lookup module in advance. In the actual work process, the target engine speed is determined based on the accelerator pedal opening signal *a*. During tractor operation, the speed error Δ*n*_*e*_ between the actual engine speed *n*_e_ and the target speed *n*_eo_, and error change rate Δ*n*’_*e*_ are inputted into the fuzzy controller, which adjusts the throttle opening *a*_*x*_ based on the working state to ensure that the engine can operate near the target speed and maintain the stability of the engine operation.

#### 2. Fuzzy controller design

*(1) Fuzzification of input and output variables*. Input variables, Δ*n*_*e*_ and Δ*n*’_*e*_, are described in fuzzy language as {negative big, negative medium, negative small, zero, positive small, positive medium, positive big}, corresponding to the fuzzy subsets {NB, NM, NS, O, PS, PM, PB}. The basic domains of Δ*n*_*e*_ and Δ*n*’_*e*_ are set to [-0.5, 0.5] and [-0.1, 0.1], respectively. To ensure that each fuzzy subset adequately covers the domain and avoid uncontrolled situations, the fuzzy domains of Δ*n*_*e*_ and Δ*n*’_*e*_ are set to {-5, -4, -3, -2, -1, 0, 1, 2, 3, 4, 5} and {-6, -5, -4, -3, -2, -1, 0, 1, 2, 3, 4, 5, 6}, respectively. Based on the basic and fuzzy domains, the input quantization factors *f*_1_ = 5/0.5 = 10, and *f*_2_ = 6/0.1 = 60.

The output variable, throttle opening *a*_*x*_, is described in fuzzy language as {negative big, negative medium, negative small, zero, positive small, positive medium, positive big}, corresponding to the fuzzy subsets {NB, NM, NS, O, PS, PM, PB}. The basic domain of ax is set to [-1, 1]. Similarly, to ensure that each fuzzy subset adequately covers the domain, the fuzzy domain of *a*_*x*_ is set to {-6, -5, -4, -3, -2, -1, 0, 1, 2, 3, 4, 5, 6}. The quantization factor for the output variable *a*_*x*_ is calculated as *f*_*3*_ = 1/6 = 0.167.

**Fig 7:**
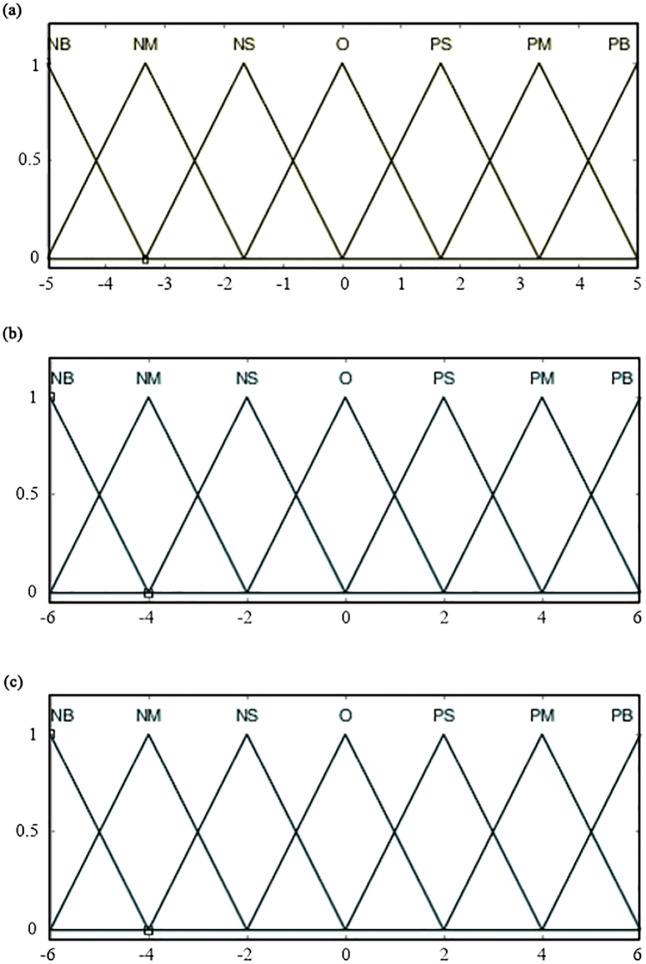
Membership functions of input and output variables. (a) *Δn*_*e*_; (b) Δ*n*’_*e*_; (c) *a*_*x*_.

*(2) Membership functions of fuzzy subsets*. Both input and output fuzzy subsets use trimf membership functions. The membership functions for Δn_e_, Δ*n*’_*e*_ and *a*_*x*_ are illustrated in Fig 7.

*(3) Fuzzy control rules*. To ensure that the engine can operate stably near the target speed. The developed fuzzy control rules for engine speed are shown in Table 2.

**Table 2:** Fuzzy control rule table of engine speed.

*a*_x_		Δ*n*’_*e*_						
		PB	PM	PS	O	NS	NM	NB
Δ*n*_*e*_	PB	PB	PB	PB	PB	PB	PM	PM
	PM	PB	PB	PM	PM	PM	PM	PM
	PS	PM	PM	PS	PS	PS	PS	PS
	O	NS	NS	O	O	O	PS	PS
	NS	NM	NM	NS	NS	NS	NS	NS
	NM	NB	NB	NM	NM	NM	NM	NS
	NB	NB	NB	NB	NB	NB	NM	NM

**Fig 8:**
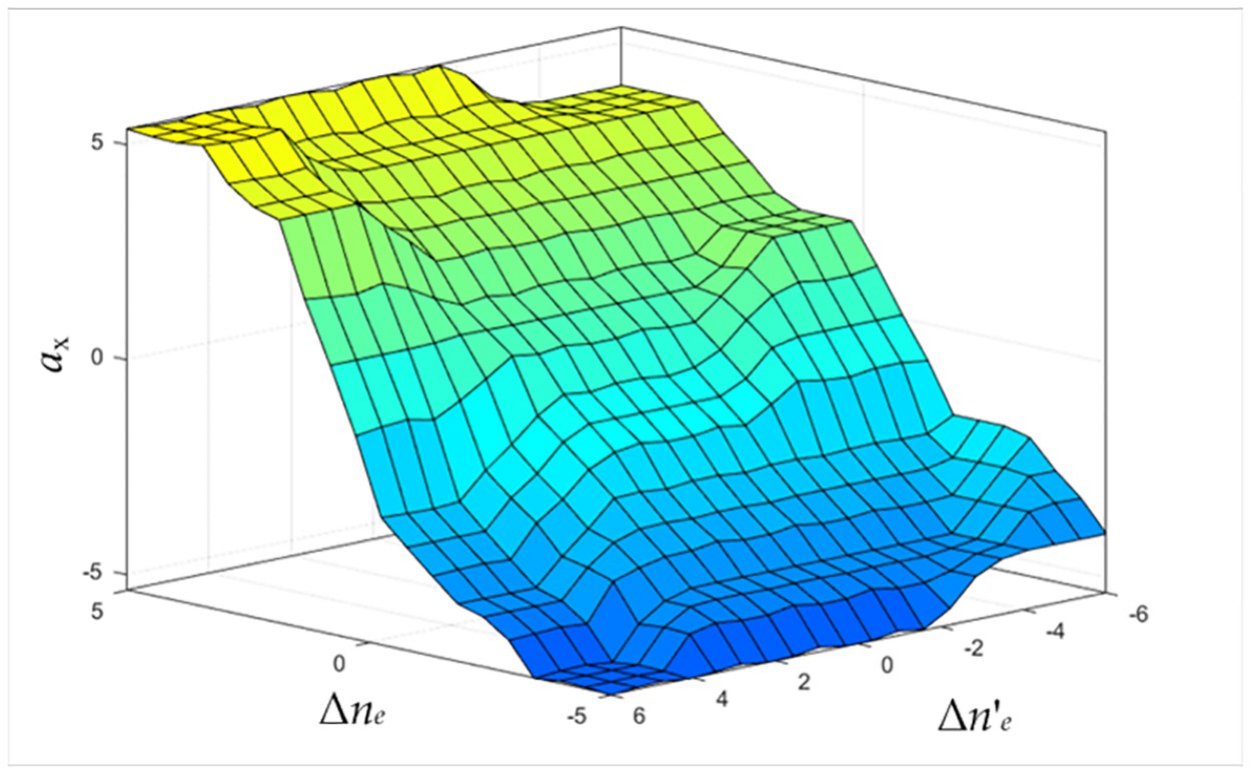
Output surface of fuzzy controller for engine speed.

The output surface of the engine speed fuzzy controller obtained by fuzzy inference on input variables is shown in Fig 8.

### Displacement ratio control

**Fig 9:**
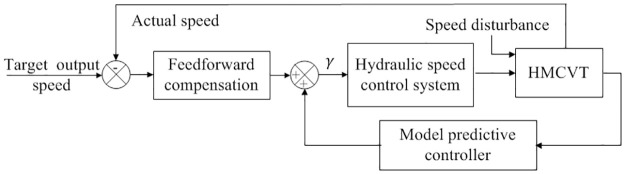
Control scheme based on feed-forward compensation.

The key to controlling the output speed of HMCVT is to control the displacement ratio of the hydraulic speed control system, that is, to adjust the swash plate swing angle value of the variable pump. Therefore, an optimal control scheme based on feedforward compensation and model predictive control is proposed, as shown in Fig 9.

Real time adjustment of speed disturbance is achieved through the feedforward compensation control part to reduce the dynamic time delay of the hydraulic speed control system and improve the response speed. Combining model prediction control to adjust the swash plate swing angle value, reduce the fluctuation of HMCVT output speed, and improve the stability of tractor operation speed.

#### 1. Hydraulic speed control system model

**Fig 10:**
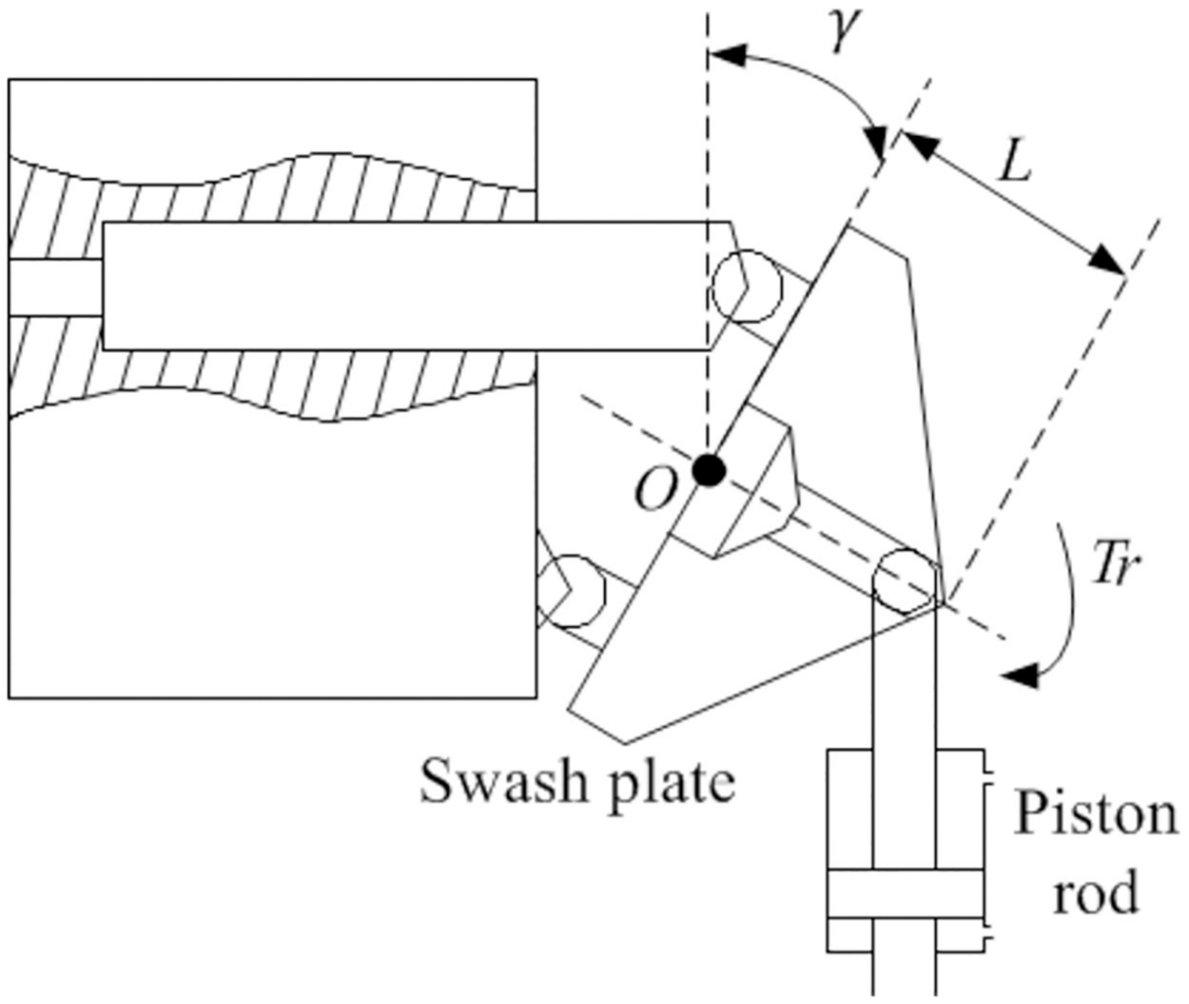
Schematic diagram of swash plate control mechanism.

The hydraulic speed control system is composed of a variable displacement pump and a fixed displacement motor. The control mechanism schematic diagram of the variable displacement pump swash plate is shown in Fig 10. The swash plate is hinged with the piston rod of the hydraulic cylinder, and the piston rod moves up and down under pressure, thereby driving the swash plate to rotate around point O. By changing the swash plate swing angle value to change the working volume of the variable displacement pump, the displacement ratio can be adjusted.

The relationship between the swash plate swing angle of the variable displacement pump and the displacement of the hydraulic cylinder piston is:

$$x_{\text{p}}=L\;\text{sin}\;\gamma$$ (33)

Where *γ* is the swash plate swing angle of the variable displacement pump, *x*_*p*_ is the piston displacement, *L* is the distance from the hinge point of the swash plate and hydraulic cylinder piston rod to the O point of the swash plate.

The swash plate swings at a relatively small angle during operation, with a general angle range of -18°-18°, sinγ can be approximated as γ. The swash plate swing angle can be calculated by the following equation:

$$\gamma =\frac{x_{\text{p}}}{L}$$ (34)

The displacement ratio *e* can be expressed as the swash plate swing angle value:

$$e=\frac{D_{\text{p}}}{D_{\text{Pmax}}}=D_{\text{Pmax}}\cdot \frac{\gamma }{\left|\gamma_{\text{max}}\right|}$$ (35)

The displacement equation of the variable displacement pump is:

$$D_{p}=k_{pm}\gamma$$ (36)

Where *k*_*pm*_ is the displacement gradient of variable displacement pump.

The continuous flow equation of the hydraulic speed control system is:

$$k_{pm}\gamma \omega_{p}=D_{m}\omega_{m}+2C_{t}\Delta P+\frac{V_{0}\text{d}\Delta P}{\beta_{\text{e}}\text{d}t}$$ (37)

Where *C*_*t*_ is the total leakage coefficient, *β*_*e*_ is the elastic modulus of the oil, *V*_0_ is the total working volume, *ω*_p_ is the angular velocity at the input end of the variable displacement pump, *ω*_m_ is the angular velocity at the output end of the motor, Δ*P* is the working pressure of the hydraulic speed control system.

#### 2. Feedforward compensation control

Feedforward compensation converts the speed disturbance of the hydraulic speed control system into real-time compensation for the swash plate swing angle of the variable pump. The engine speed error and system leakage of the hydraulic speed control system are taken into consideration. A formula for the compensation value *γ*_o_ of the variable pump swash plate angle is derived.

$$\gamma_{o}=\frac{D_{\text{p}}\Delta n_{e}}{2\pi k_{pm}i_{1}i_{2}}-\frac{C_{t}P_{h}}{k_{pm}\omega_{p}}$$ (38)

Where *P*_*h*_ is the high-pressure side pressure of the hydraulic speed control system.

#### 3. Model predictive control

**Fig 11:**
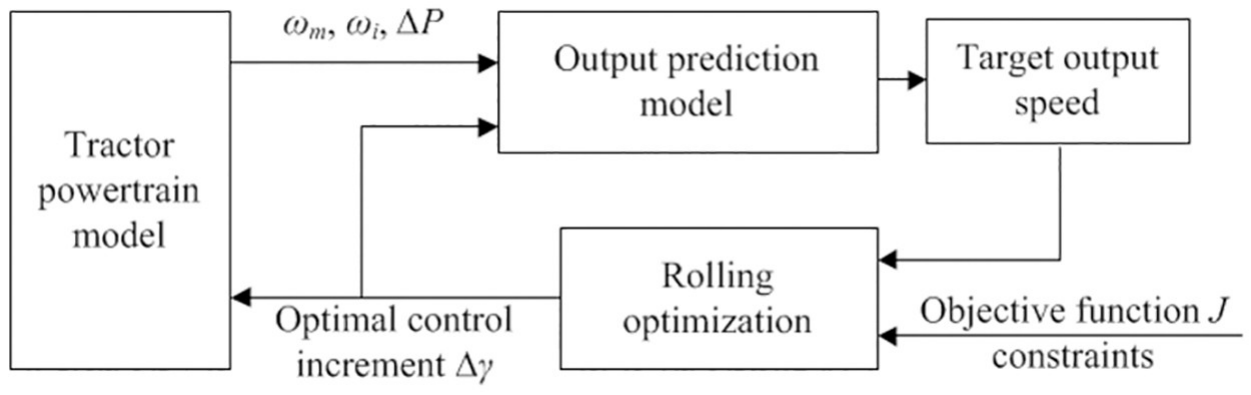
Model prediction control principle.

Model predictive control is a decision-making process based on feedback information, and its control principle is shown in Fig 11.

The model predictive controller predicts the future output of HMCVT based on the current state information. By comparing it with the target speed and combining it with the objective function, the optimal control increment Δ*γ* for the next time is solved. The increment is applied to the variable pump swash plate, and the above process can be repeated to rolling solve the future swash plate swing angle value, achieving the model predictive optimal control.

*(1) State space equation of HMCVT*. According to the control strategy formulated, take the motor angular velocity *ω*_m_, HMCVT input shaft angular velocity *ω*_*i*_ = *ω*_*e*_, the working pressure ΔP as the state variable of the system. Select the swash plate swing angle *γ* as the control variable. d is measurable disturbances of HMCVT. The state space equation for optimizing the control of the swash plate swing angle of the variable displacement pump can be obtained as follows:

$$\left\{\begin{array}{l} \dot{x}=Ax+Bu+Fd\\ y=Cx \end{array}\right.$$ (39)

$$A=\left(\begin{array}{ccc} -\frac{b_{r1}+b_{c2}+b_{o}}{J_{r1}+J_{c2}+J_{o}} & 0 & 0\\ 0 & -\frac{b_{i}}{J_{i}} & 0\\ 0 & 0 & -\frac{2C_{t}\beta_{e}}{V_{0}} \end{array}\right)\;B=\left(\begin{array}{c} 0\\ 0\\ \frac{k_{pm}\omega_{p}\beta_{e}}{V_{0}} \end{array}\right)$$

$$F=\left(\begin{array}{ccc} \frac{1}{J_{r1}\text{+}J_{\text{c}2}+J_{0}} & 0 & 0\\ 0 & \frac{1}{i_{1}i_{2}J_{i}} & 0\\ 0 & 0 & -\frac{D_{m}\beta_{e}}{V_{0}} \end{array}\right)\;C={\left(\begin{array}{c} 1\\ 0\\ 0 \end{array}\right)}^{\text{T}}\;d=\left(\begin{array}{c} T_{r1}+T_{c2}-T_{o}\\ (T_{i}-T_{c1})i_{1}i_{2}-T_{p}\\ \omega_{m} \end{array}\right)$$

Where A, B, F and C are the coefficient matrices of HMCVT’s state variables, control variables, measurable disturbances, and output variables, respectively.

Discretize Eq 39 using the first-order difference quotient method, with a sampling time T. To reduce or eliminate static errors, rewrite the discretization equation into incremental equation:

$$\left\{\begin{array}{l} \Delta x(k+1)=A_{x}\Delta x(k)+B_{u}\Delta u(k)+F_{d}\Delta d(k)\\ \Delta y(k)=C\Delta x(k) \end{array}\right.$$ (40)

Δx(k)=x(k)−x(k−1)

Δu(k)=u(k)−u(k−1)

Δd(k)=d(k)−d(k−1)

$$A_{x}=e^{AT},B_{u}=\int_{0}^{T}e^{At}\text{d}t\cdot B,\;F_{d}=\int_{0}^{T}e^{At}\text{d}t\cdot F$$ (41)

Where Δ*x*(*k*) is the state increment, Δ*u*(*k*) is the control input increment, Δ*d*(*k*) is the measurable interference increment, Δ*y*(*k*) is the controlled output increment, *A*_*x*_, *B*_*u*_ and *F*_*d*_ are the coefficient matrices of discretized state variables, control variables, and measurable disturbance, respectively.

*(2) Output prediction model*. According to the principle of model predictive control, the latest measured value at the current moment is taken as the initial condition, and the future value is predicted based on the output predictive model. Due to the principle that the control time domain is smaller than the prediction time domain, it is assumed that the control interval and prediction interval are *M*_*c*_ and *M*_*p*_, with *M*_*c*_≤*M*_*p*_, respectively; After the interval k, the measurable interference remains unchanged, Δd(k+i) = 0, i = 1,2,⋯,Mp-1. The output prediction model can be derived from Eq 40 as follows:

$$Y(k+1)=S_{x}\Delta x(k)+y(k)+S_{d}\Delta d(k)+S_{u}\Delta U(k)$$ (42)

Where

$$\Delta U={\left(\begin{array}{cccc} \Delta u(k) & \Delta u(k+1) & \cdots & \Delta u(k+M_{c}-1) \end{array}\right)}^{Τ}$$

$$Y(k+1)={\left(\begin{array}{cccc} y(k+1\left|k)\right. & y(k+2\left|k)\right. & \cdots & y(k+M_{P}\left|k)\right. \end{array}\right)}^{Τ}$$

$$S_{x}={\left(\begin{array}{cccc} CA_{x} & \sum_{i=1}^{2}CA_{x}^{2} & \cdots & \sum_{i=1}^{M_{p}}CA_{x}^{i} \end{array}\right)}_{1\times M_{p}}^{Τ}$$

$$S_{d}={\left(\begin{array}{cccc} CF_{d} & \sum_{i=1}^{2}CF_{d}^{i} & \cdots & \sum_{i=1}^{M_{p}}CF_{d}^{i} \end{array}\right)}_{1\times M_{p}}^{Τ}$$

$$S_{u}={\left(\begin{array}{cccc} CB_{u} & 0 & \cdots & 0\\ \sum_{i=1}^{2}CA_{x}^{i-1}B_{u} & CB_{u} & \cdots & 0\\ \vdots & \vdots & \ddots & \vdots \\ \sum_{i=1}^{M_{p}}CA_{x}^{i-1}B_{u} & \sum_{i=1}^{M_{p}-1}CA_{x}^{i-1}B_{u} & \cdots & \sum_{i=1}^{M_{p}-M_{c}+1}CA_{x}^{i-1}B_{u} \end{array}\right)}_{M_{p}\times M_{c}}$$ (43)

Where *M*_*p*_ is the prediction interval; *M*_*c*_ is the control interval; *S*_*x*_, *S*_*d*_ and *S*_*u*_ are the state variables, control variables, and measurable interference coefficient matrices of the output prediction model, respectively.

*(3) Objective function*. The purpose of designing model predictive controller is to enable the output speed of the HMCVT to more accurately and steadily follow the target speed. Therefore, while controlling the output speed to quickly approach the reference trajectory, the amplitude of the control variable cannot be too large. In the objective function, the influence of the current control *u*(*k*) on the future time of the system is considered, and the objective function is adopted as follows:

$$H=\sum_{i}^{M_{P}}{\left\|y(k+i)-y_{ref}(k+i)\right\|}_{Q}^{2}+\sum_{i}^{M_{C}}{\left\|\Delta u(k+i-1)\right\|}_{R}^{2}$$ (44)

Where *Q* is the weight coefficient matrix of state error; *R* is the matrix of control increment weight coefficients; *y*(*k+i*) is the predicted output shaft speed; *y*_*ref*_(*k+i*) is the reference sequence.

For ease of calculation, the objective function *H* of the model predicted controller is transformed into a standard quadratic programming form as follows:

$$J=U{\left(k\right)}^{Τ}({S_{u}}^{Τ}QS_{u}+R)U(k)+2E^{Τ}QS_{u}U(k)$$ (45)

$$E=S_{x}\Delta U(k),\;\left(\begin{array}{c} I\\ -I \end{array}\right)\Delta U(k)\leq \left(\begin{array}{c} \Delta u_{\text{max}}\\ \Delta u_{\text{min}} \end{array}\right)$$ (46)

Where, *I* is the unit coefficient matrix; *Δu*_max_ and *Δu*_min_ represent the boundaries of the maximum and minimum control increments, respectively.

Within the control interval, based on the latest measurement state information of HMCVT, the model predictive controller obtains a column of optimal control increments ΔU*(*k*) for each rolling search for the optimal solution based on the objective function. The first term is taken as the input for the swash plate swing angle of variable pump, and the optimal control quantity at time *k* is:

$$u(k)=u(k-1)+\left(\begin{array}{cccc} I & 0 & \cdots & 0 \end{array}\right)\Delta U^{*}(k)$$ (47)

## Simulation analysis

### Vehicle simulation model

**Fig 12:**
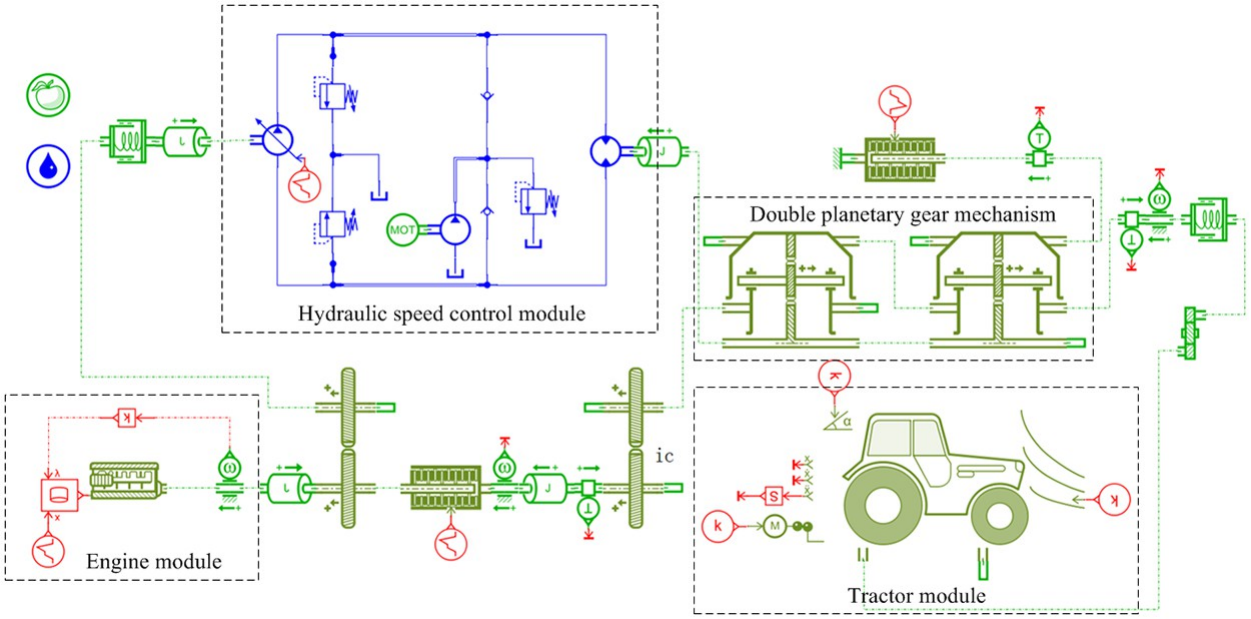
Tractor simulation model.

A complete tractor simulation model was established based on AMEsim, as shown in Fig 12. It mainly consists of modules such as engine, hydraulic speed control system, planetary gear, and tractor.

The mathematical model of tractor operation resistance:

$$F_{t}=k_{t}b_{t}h_{t}z_{t}$$ (48)

Where, *F*_*t*_ is the tractor traction resistance, *k*_*t*_ is the soil specific resistance, *b*_*t*_ is the width of the plowshare, *h*_*t*_ is the plowing depth, *z*_*t*_ is the number of plowshares.

When the tractor performs plowing operations, the following resistance balance conditions are met:

$$F_{f}=F_{t}+fG+G\;\text{sin}\;\theta$$ (49)

Where *F*_*f*_ is the resistance of the tractor during plowing operations, *G* is the total weight of the tractor, *f* is the comprehensive resistance coefficient, *θ* is the slope of the tractor operation.

**Fig 13:**
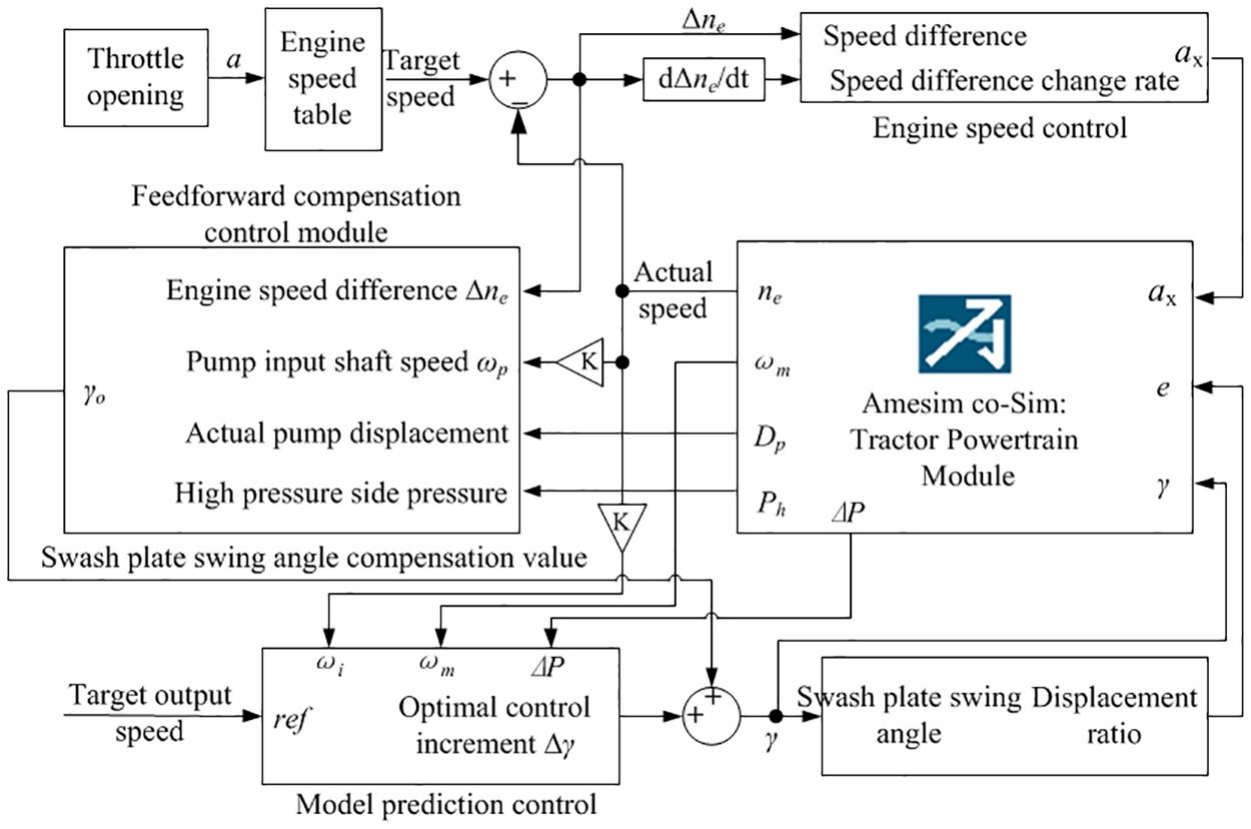
Co-simulation model of HMCVT output speed control.

The joint simulation model of HMCVT output speed control based on the AMEsim and Simulink is shown in Fig 13. It includes the tractor powertrain module, engine speed control module, throttle opening acquisition module, model prediction control module, and feedforward compensation control module. For the collection of throttle opening signals, a lookup table module was used. This module provides accurate target engine operating speed based on a preset relationship table between throttle opening and engine speed.

The basic simulation parameters are shown in Table 3.

**Table 3:** Basic parameters of the simulation model.

Parameter	Values	Parameter	Values
Drive wheel radius /m	0.346	Hydraulic motor displacement /mL·r^-1^	32
Main reducer speed ratio	21.32	Maximum displacement of variable displacement pump /mL·r^-1^	45
Characteristic parameters of planetary row 1	4	Variable pump speed range/r·min^-1^	0~1800
Characteristic parameters of planetary row 2	1.5	Hydraulic motor speed range /r·min^-1^	0~1800
Pressure setting of relief valve /MPa	40	Rotational inertia of hydraulic motor /kg·m^2^	0.9
Total leakage coefficient of hydraulic system /*m*^2^·(*s*·*Pa*)^-1^	4.91×10^−12^		

### Simulation model validation

**Fig 14:**
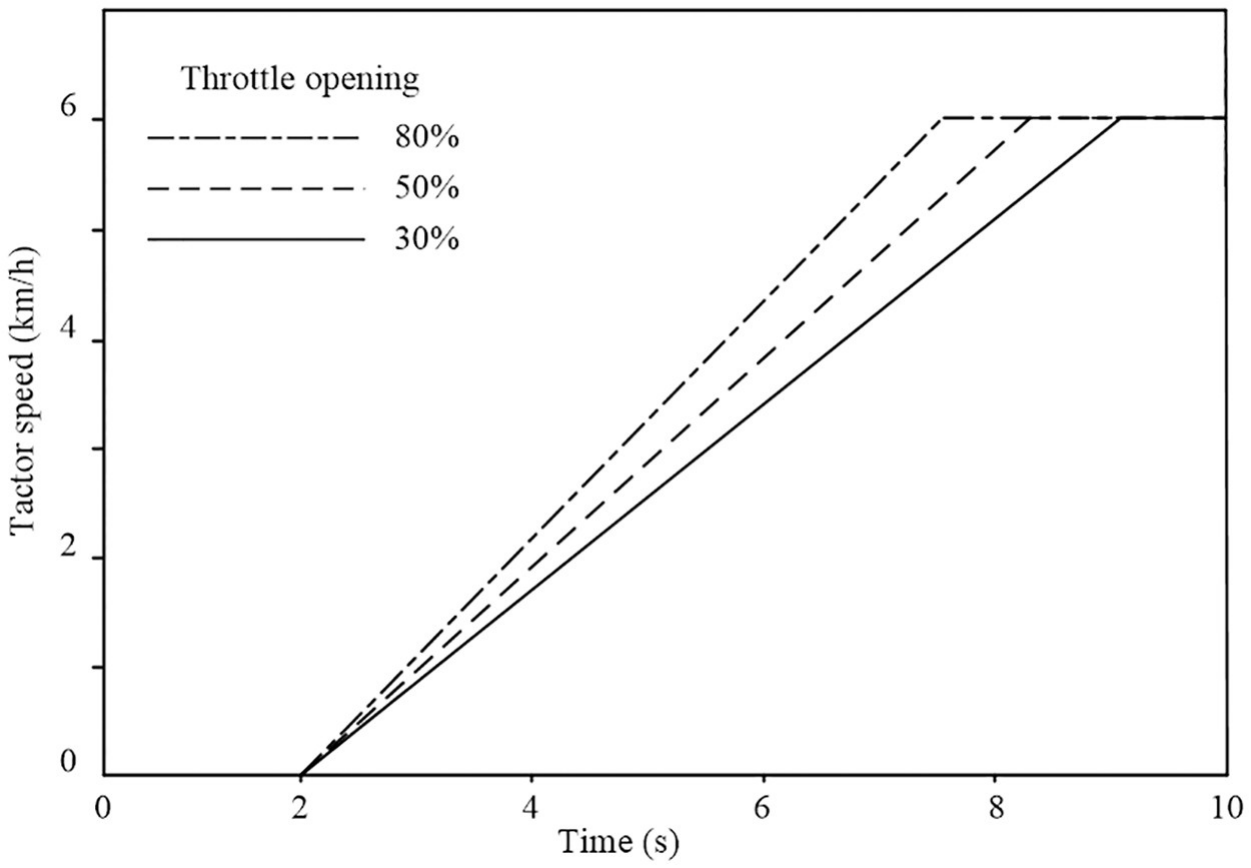
Tractor speed regulation simulation curve.

To verify the correctness of the simulation model, speed control simulation tests were conducted on the constructed whole vehicle model in AMEsim. During simulation, HMCVT is in hydraulic mechanical mode HM2, and the engine throttle opening is set to 30%, 50%, and 80% respectively. The displacement ratio of hydraulic speed control system is adjusted from 0 to 0.6. The tractor speed simulation curve is shown in Fig 14.

It can be seen that during the operation of the tractor, by adjusting the engine throttle opening and the displacement ratio of variable pump, the tractor’s speed and speed ratio will gradually increase with displacement ratio. Compared with the theoretical curve of HMCVT speed regulation characteristics analyzed in Section 2.2.1, the curve changes are consistent. This verifies the correctness of the whole vehicle simulation model built in this article.

### Simulation analysis

To explore the effectiveness of the proposed comprehensive control strategy for HMCVT output speed in this article, a comparative simulation was conducted using the strategy and PID control method for accelerated working mode and load step change mode. The simulation conditions are: the tractor performs field plowing operations, with a plowshare width of 22cm, 6 plowshares, a total weight of 40600N for the tractor and plow tools, a comprehensive resistance coefficient of 0.03, a soil specific resistance of 5 N/cm^2^, and operation slope of 0°.

#### Simulation 1

**Fig 15:**
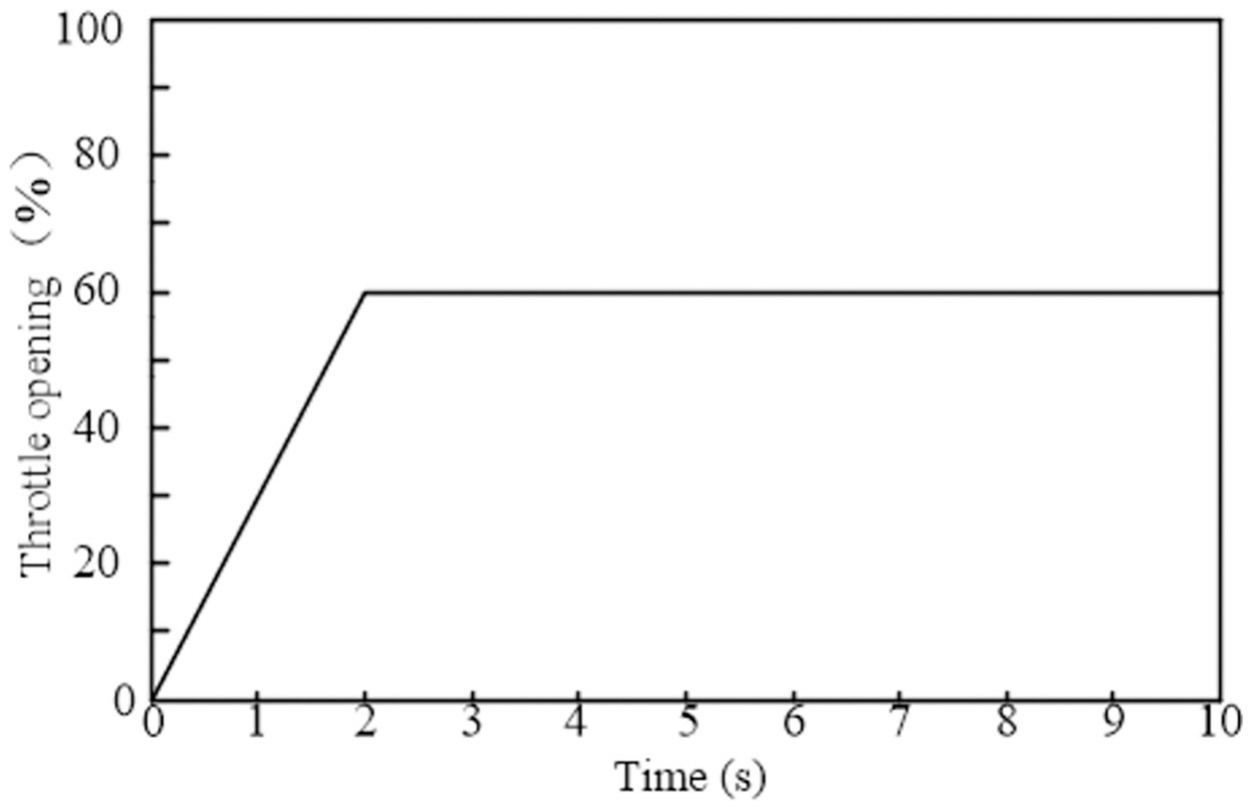
Change of throttle opening.

The working conditions of the tractor are complex and variable, especially when working in the field. The tractor faces frequent starting and stopping problems. The prerequisite for efficient operation of the tractor is to be able to start normally. Therefore, for the analysis of the tractor acceleration mode, the starting stage is selected, and the throttle opening is set as shown in Fig 15.

**Fig 16:**
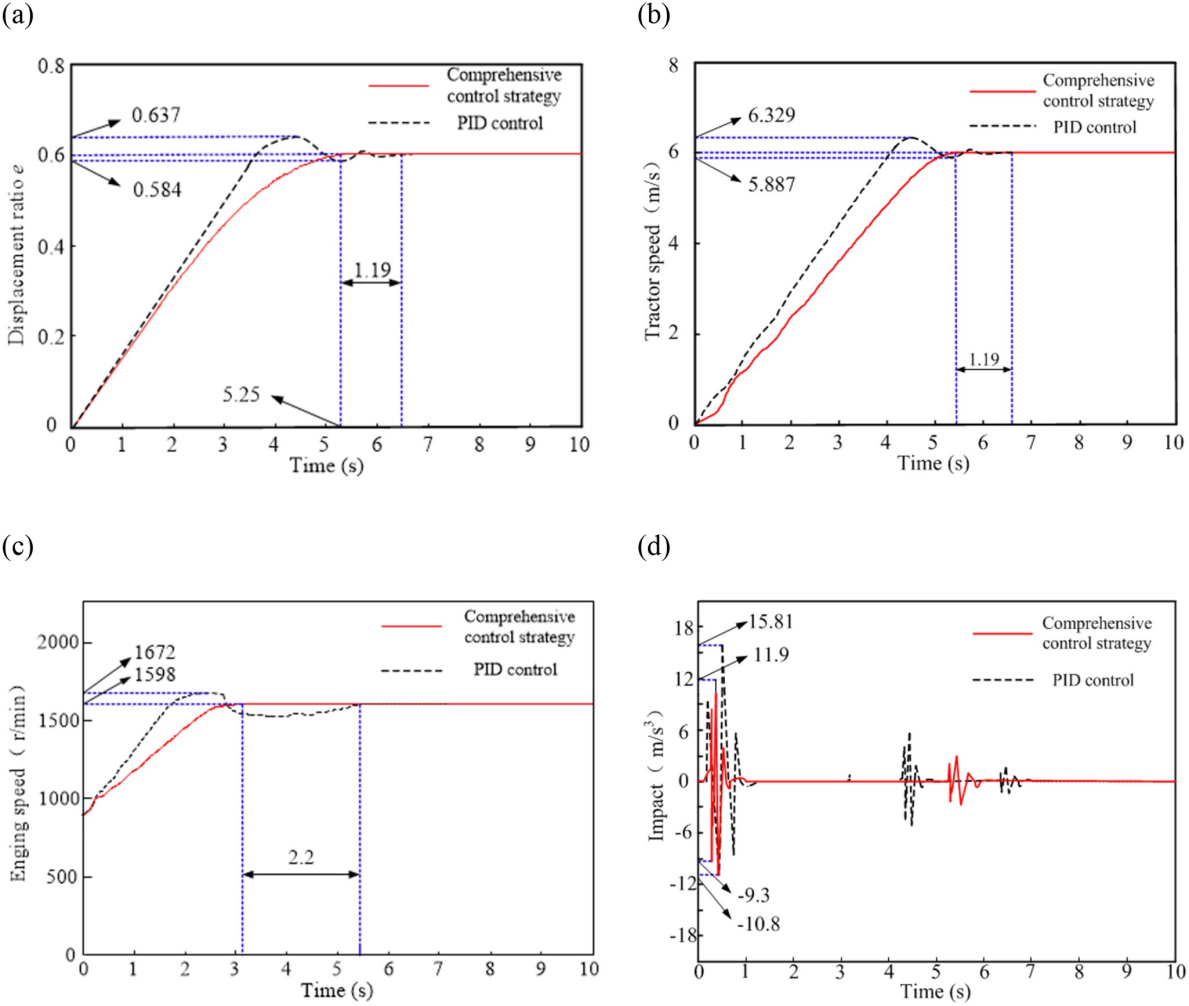
Simulation results of tractor starting process.

When the tractor starts, HMCVT is in pure hydraulic start mode H. Based on the constructed joint simulation model, the simulation results of the tractor starting are shown in Fig 16.

Fig 16(a) shows the simulation comparison results of the displacement ratio. When using the control strategy developed in this paper, the tractor’s starting process is relatively slow compared to using PID control to achieve the target displacement ratio. But when using PID control, there is an overshoot in the displacement ratio, with a fluctuation rate of 8.83%. Some working sections of the system are in an unstable state. When using the comprehensive control strategy, the displacement ratio reaches stability 1.19 seconds earlier, which improves the stability of the HMCVT output speed when the tractor starts.

Fig 16(b) shows the comparison simulation results of tractor speed. It can be seen from the figure that when using PID control, the speed to reach the target tractor speed is faster, but there is a significant speed fluctuation, with a fluctuation rate of 7.36%; When using the comprehensive control strategy, there is no overshoot phenomenon. The time for the tractor to reach a stable speed has been reduced by 18.5%, which proves that this control strategy can effectively improve the stability of HMCVT output speed and reduce the starting time of the tractor.

Fig 16(c) shows the comparison simulation results of engine speed. When using comprehensive control strategy, the time for the engine speed to reach the target speed for stable operation is 41.6% earlier than using PID control. The fluctuation rate of engine speed is reduced by 8.9%, which improves the stability of engine operation.

Fig 16(d) shows the comparison results of impact simulation, where the impact changes significantly during the initial stage of vehicle speed increasing from zero. For tractors using comprehensive control strategy, the maximum impact when starting is 11.9m/s^3^, which is 24.7% lower than the 15.81m/s^3^ when using PID control. The control strategy developed in this article improves the smoothness of tractor starting.

Based on the above analysis, it can be concluded that the comprehensive control strategy formulated in this article can achieve good control effects when the tractor is in the starting condition, and enhance the stability of HMCVT output speed.

#### Simulation 2

During the operation of the tractor, the load resistance is often in an unstable state, causing fluctuations in the output speed and engine speed. Therefore, it is necessary to use the step change working condition of tractor operation resistance to simulate and analyze the control effect.

**Fig 17:**
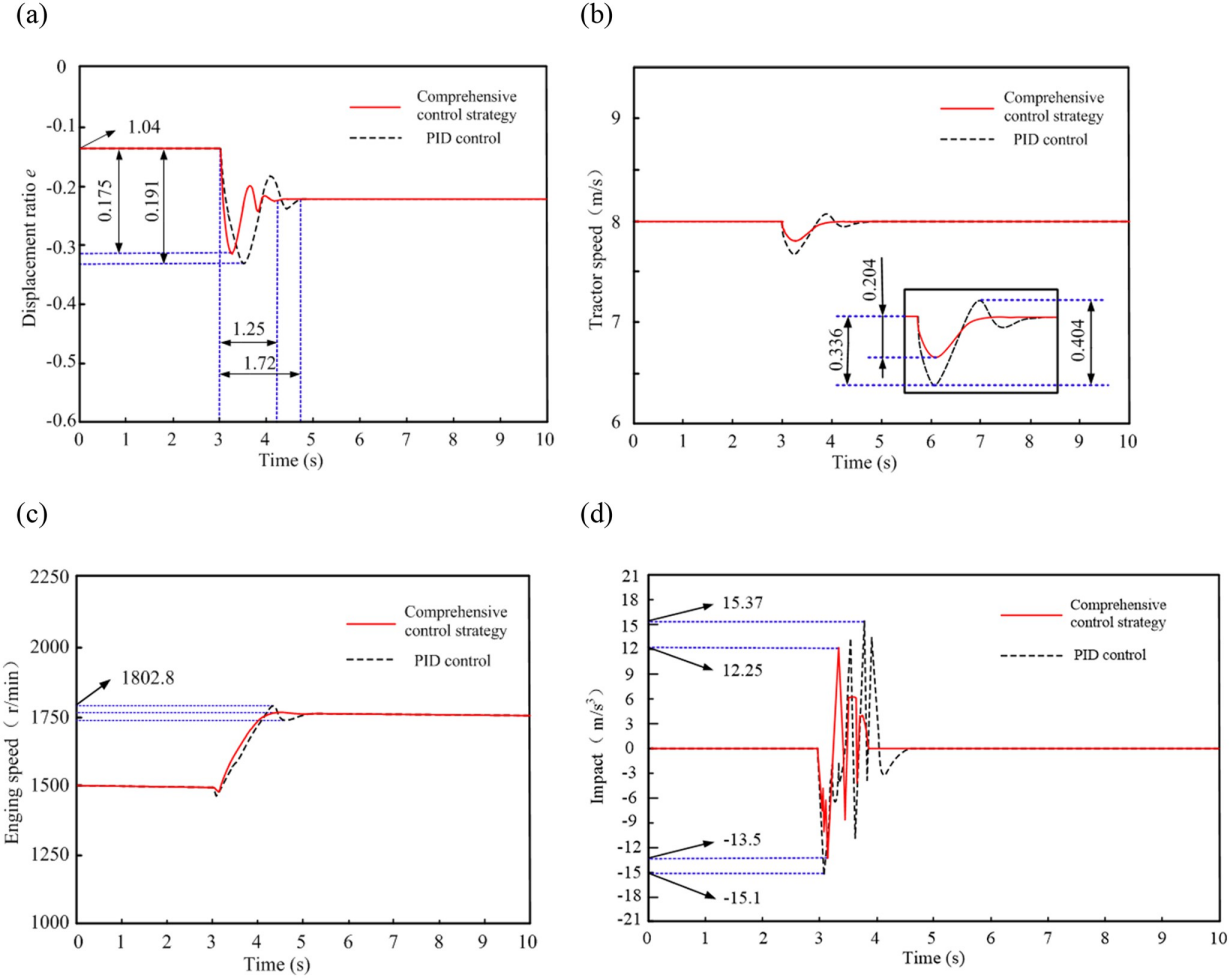
Simulation results of the steady speed process of tractor step load.

The simulation conditions are set as follows: the tractor is engaged in plowing operation, without considering the influence of slip rate on tractor speed. The HMCVT is in hydraulic mechanical mode HM1, with a displacement ratio range of 0.8~-0.6. The initial plowing depth of the tractor is 10cm, and the operating speed is stable at 8km/h. In the third second, the plowing depth steps to 20cm, and the tractor’s load resistance steps from 6kN to 14kN. The simulation time is 10s. According to the set simulation conditions, the step load disturbance process of the tractor was simulated and analyzed, and the simulation results are shown in Fig 17.

Fig 17(a) shows the comparison results of displacement ratio. When the tractor is subjected to step load disturbance, the comprehensive control method designed in this paper is adopted. The displacement ratio of the variable pump is reduced to compensate for the output speed fluctuation caused by load resistance disturbance speed, with a maximum reduction of 0.175. The variable pump swash plate responds quickly and the adjustment time is 1.25 seconds; Using the PID method, the maximum reduction in the swash plate swing angle of the variable pump is 0.191, and the adjustment time is 1.72 seconds; Compared with PID control, the comprehensive control method is more sensitive to step disturbance response of load resistance.

Fig 17(b) shows comparison results of tractor speed. When using PID control, the speed to reach the target tractor speed is faster, but there is a significant fluctuation; When adopts the comprehensive control method, the speed fluctuation rate reduces 2.5% and the adjustment time shorten 0.47 seconds. This strategy improves the stability of tractor speed and reduces the speed adjustment time.

Fig 17(c) shows the comparison results of engine speed. During the stable speed process of tractor, the engine speed under both control methods showed a decrease due to step load disturbance. The engine speed response was faster when using the comprehensive control method, and the fluctuation rate of engine speed decreased by 3.52% compared to PID control strategy.

Fig 17(d) shows the comparison results of impact. Under the PID control method, the impact during the stable speed process of the tractor is between (-13.5m/s^3^, 12.25m/s^3^). The comprehensive control strategy controls the impact between (-15.1m/s^3^, 15.37m/s^3^). Compared with the PID method, the maximum impact is reduced by 12.2%.

The above simulation results shows that when HMCVT is disturbed by step load resistance, the optimal control method based on feed forward compensation effectively improves the stability of HMCVT, and its control effect is significantly better than traditional PID method.

## Conclusion

The output speed control strategy that integrates engine speed control and the displacement ratio control of HMCVT is investigated. The tractor starting and step load disturbance processes are simulated and discussed. The results suggest the following.

1. The structure and the characteristics of three-stage HMCVT suitable for tractor were analyzed, including speed regulation characteristics, torque characteristics, and power diversion characteristics. The results indicate that the HMCVT has the speed characteristic of stepless speed regulation from zero, and the hydraulic power split ratio is controlled within 0.4, which can ensure the efficiency of tractor transmission.
2. A comprehensive output speed control strategy combining engine speed control and hydraulic speed control system displacement ratio control has been developed to address the issue of fluctuations in the output speed of HMCVT. Identify changes in load resistance through fuzzy control and control the engine speed to maintain the maximum output torque corresponding to each throttle opening at the corresponding speed; Real time adjustment of speed disturbance is achieved through feed forward compensation, combined with model prediction control to adjust the swash angle of the variable pump. This improves the anti-interference ability and output speed stability of tractors equipped with HMCVT during operation.
3. A joint simulation platform was established based on AMEsim and Simulink, and simulation analysis was conducted on the tractor acceleration mode and load step mode. The results show that compared with PID control, using the comprehensive control strategy developed in this paper, under the load step mode, the maximum impact is reduced by 12.2%, the tractor speed fluctuation rate is reduced by 2.5%, and the adjustment time is shortened by 0.47 seconds, which improved the stability of the HMCVT output speed.

## Supporting information

S1 File Information on engine model and output speed stability evaluation index. (PDF)
