# Supplementary material for: Output speed control for hydro-mechanical continuously variable transmission of tractor
Source: PLoS One. 2024 Sep 24;19(9):e0308493. doi: 10.1371/journal.pone.0308493 (PMC11421825; doi:10.1371/journal.pone.0308493)
Supplement: S1 File — (PDF) [file pone.0308493.s001.pdf]

1. This article selects the LR6105ZT10 engine model and establishes a mathematical model of the engine based on the universal characteristic test data conducted on the engine.

When building the simulation model, the torque and speed data of engine test is imported into AMESim's engine model and compiled into an ASCII data file, which is then input into the asciifoficy module to establish the engine simulation model.

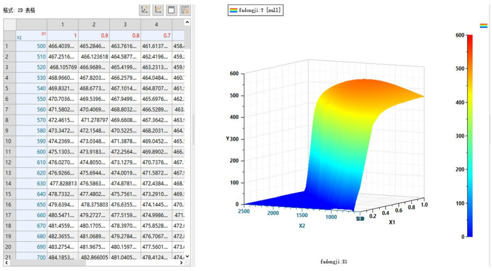

Fig. 1 Engine simulation model

2. HMCVT output speed stability evaluation index

The stability evaluation index of HMCVT output speed can intuitively reflect the stability of HMCVT output speed in numerical form. This article uses the impact degree and the fluctuation rate of tractor HMCVT output speed to reflect the quality of HMCVT speed regulation process.

(1) Impact degree

Impact degree is the first-order derivative of tractor acceleration, reflecting the driver's subjective perception of vehicle smoothness. The expression is as follows:

$$j = \frac{da}{dt} = \frac{d^2 v}{dt^2} = \frac{\pi r_c}{i_0}\frac{d^2 n_0}{dt^2} = \frac{k_2 \pi r_c}{(1+k_2) i_0}\frac{d^2 \omega_m}{dt^2} \qquad (1)$$

Where, j is the impact degree, m/s3; a is the longitudinal acceleration speed of the tractor, m/s2; i_0 is the transmission ratio of the main reducer; r_c is the radius of the driving wheel, m.

(2) HMCVT output speed fluctuation rate

The output speed fluctuation rate refers to the ratio of the difference between the maximum and minimum output speed of the tractor HMCVT to the steady-state rotational speed, reflecting the stability of the tractor HMCVT system. The smaller the fluctuation rate, the more stable it is. Its expression is:

$$\sigma = \frac{n_{\max} - n_{\min}}{\overline{n}} \times 100\% \qquad (2)$$

Where, σ is the output rotational speed fluctuation rate of the tractor HMCVT; $\overline{n}$ is the stable output rotational speed of the tractor HMCVT, r/min; $n_{\max}$ is the maximum output rotational speed during the speed regulation process of the tractor HMCVT, r/min; $n_{\min}$ is the minimum output rotational speed during the speed regulation process of the tractor HMCVT, r/min.
